# Correction to: Risk of stress/depression and functional impairment in Denmark immediately following a COVID-19 shutdown

**DOI:** 10.1186/s12889-021-11204-x

**Published:** 2021-06-16

**Authors:** Lars H. Andersen, Peter Fallesen, Tim A. Bruckner

**Affiliations:** 1 ROCKWOOL Foundation, Ny Kongensgade 6, 1472 Copenhagen C, Denmark; 2 Swedish Institute for Social Research, Stockholm University, 106 91 Stockholm, Sweden; 3 Program in Public Health and the Center for Population, Inequality and Policy, University of California, Irvine, CA 92697-3957 USA

**Correction to: BMC Public Health 21, 984 (2021)**

**https://doi.org/10.1186/s12889-021-11020-3**

It was highlighted that the original article [1] contained some errors in the references. The title of reference [2] was incorrect and a new reference had to be added and cited in the Discussion section of the text. This correction article shows the correct reference [2], the new reference [3] and its in-text citation. The reference list in the text has been renumbered from reference [3] onwards. The original article has been updated.

**Discussion p.8**

Denmark, although mental wellbeing (also measured using the WHO-5) in that study is observed only after the lockdown, from early to late April (i.e., that study has no pre lockdown "baseline" but still documents improved mental wellbeing from early to late April) [3].
